# How Visuo-Spatial Mental Imagery Develops: Image Generation and Maintenance

**DOI:** 10.1371/journal.pone.0142566

**Published:** 2015-11-12

**Authors:** Marina C. Wimmer, Katie L. Maras, Elizabeth J Robinson, Martin J Doherty, Nicolas Pugeault

**Affiliations:** 1 University of Plymouth, School of Psychology, Cognition Centre, Plymouth, United Kingdom; 2 University of Bath, Department of Psychology, Claverton Down, Bath, United Kingdom; 3 University of Warwick, Department of Psychology, Coventry, United Kingdom; 4 University of East Anglia, School of Psychology, Norwich, United Kingdom; 5 University of Surrey, Centre of Vision, Speech and Signal Processing, Guildford, United Kingdom; Catholic University of Sacro Cuore, ITALY

## Abstract

Two experiments examined the nature of visuo-spatial mental imagery generation and maintenance in 4-, 6-, 8-, 10-year old children and adults (*N* = 211). The key questions were how image generation and maintenance develop (Experiment 1) and how accurately children and adults coordinate mental and visually perceived images (Experiment 2). Experiment 1 indicated that basic image generation and maintenance abilities are present at 4 years of age but the precision with which images are generated and maintained improves particularly between 4 and 8 years. In addition to increased precision, Experiment 2 demonstrated that generated and maintained mental images become increasingly similar to visually perceived objects. Altogether, findings suggest that for simple tasks demanding image generation and maintenance, children attain adult-like precision younger than previously reported. This research also sheds new light on the ability to coordinate mental images with visual images in children and adults.

## Introduction

Visual mental imagery, “seeing with the mind’s eye”, is when we see an event, an object or a scene in the absence of the related visual input [1, 2]. As adults we use mental imagery ubiquitously, for example, in order to think whether we can fit the car in the parking space, or how our living room would look with rearranged furniture.

Mental imagery is particularly interesting theoretically, as it lies at the intersection of vision and memory [3]. For example, it has been shown to play a fundamental role in ambiguous figures reversal in both children and adults. Generating an image of alternative interpretations of ambiguous stimuli, such as Jastrow’s duck-rabbit [4], allows its disambiguation [5, 6, 7]. Mental imagery ability is also directly linked to true and false memory processes: Children’s faster and more accurate image generation enhances visuo-spatial recall ([8], see [9] for findings in adults]. Moreover, children’s ability to mentally transform a scene or an object and to project themselves into the past, allows episodic recall for self-generated events [10]. Mental imagery also facilitates semantic memory in children and adults [11, 12], and affects semantic false memories [13, 14] as well as false memories for implanted false events [15]. Thus, mental imagery plays a significant role in the facilitation of visual perception processes and memory performance, yet little is known how mental imagery itself develops, which is examined in the current research.

Mental imagery has also been shown to be crucial in other cognitive domains such as in reasoning and problem-solving tasks [16, 17, 18]. For example, when 3-year-old children are required to predict the path of a ball rolling through curved opaque tubes, their accuracy improves when they mentally imagine the ball’s path [17]. Similarly, using mental imagery improves 2- and 3-year-olds’ reasoning on incongruent syllogisms such as, “All sheep ride bicycles, Bill is a sheep, does Bill walk or ride a bicycle?” [18]. However, children’s use of imagery can also be disadvantageous: Children are less likely to consider alternative possibilities when it is relatively easy to imagine one of the possible outcomes [19].

In sum, research indicates that children as young as 3 years are capable of using mental imagery when verbally instructed to do so [16–18]. However, it remains unclear what the nature of these mental images is and how they develop. The current aim is to examine how well children perform on a task using their remembered visuo-spatial mental images compared to when actually seeing an object or a scene (e.g., flower in a vase), and at what age their mental images become adult-like in this regard. For example, can children generate or maintain an image of a previously seen object (e.g., vase) and coordinate this with a currently visible second object (e.g., flower)?

Research on mental imagery established a distinction between mental imagery for reproductive images (i.e. evoking images for objects or events that are already known) and transformed images (i.e. evoking images for events that have previously not been perceived, including movements and transformations of previously seen objects) [20, 21, 22]. In the current research we focus on reproductive mental images. To date, only research by Kosslyn and colleagues has systematically examined how visuo-spatial mental images are generated and maintained compared to visual perception in 5-, 8-, and 14-year-old children and adults [23]. Kosslyn et al.’s [23] measure of image generation was the time taken to retrieve an image that is stored in long-term memory. In their image generation task participants judged whether two x marks fell on a previously studied letter (e.g., “G”) in a 4 x 5 cell grid when prompted with a lowercase version (i.e., “g”). The letters differed in complexity depending on how many segments were filled in the grid (e.g., “G”–complex condition), requiring more effort to generate a mental image, to fewer segments (e.g., “L”–simple condition), requiring less effort. Their findings revealed that overall only 14-year-olds performed at adult level. Further, the youngest age group, 5-year-olds, showed no response time difference between conditions of high and low cognitive load. Performance factors may have masked younger children’s image generation abilities.

In contrast to image generation, where participants have to access information from long-term memory, image maintenance requires holding in mind an image over a particular period [23]. Kosslyn and colleagues’ image maintenance task was akin to their image generation task except that abstract patterns were studied instead of letters and had to be remembered either for 500ms or 3000ms. Participants judged whether two “x” marks fell on the previously studied pattern. All participants, even 5-year-olds, performed more accurately in a light load condition (500ms) than in a heavy load condition (3000ms) but 5-year-olds showed no response time differences between conditions.

Both tasks have some limitations for assessing image generation and maintenance abilities from a developmental angle. First, for the image generation task, relatively poor facility with the alphabet may have disadvantaged younger children. Conversely, adults and older children may have been able to use their good knowledge of letters to solve the task by simply anticipating which segments may be filled in rather than retrieving an image of the previously seen stimulusfrom. Second, both tasks implemented abstract stimuli that are unlike everyday life mental imagery contexts. Third, in Kosslyn et al.’s [23] tasks encoding time varied between participants, because they indicated when they remembered the according stimulus (image maintenance) or studied letters until they reached criterion (image generation). This variance in encoding time poses two issues. First, making a correct metamemorial judgement, that is, the ability to introspect into one’s own memory processes, may have been difficult for 5-year-olds as metamemory research indicates that only children who are 8 years of age and older use metamemorial strategies successfully [24]. Second, encoding time may affect later image generation and maintenance performance. That is, extended imagery time leads to stronger effects of imagery on subsequent binocular rivalry [25]. Moreover, if mental imagery facilitates memory performance [8–11] then the question occurs whether this relation is bidirectional and memory capacity directly interferes with a potential use of imagery in children. Therefore, performance differences across participants may have been the result of variations in encoding time rather than directly reflecting variations in imagery ability *per se*. Finally, the 6-year gap between the 8-year-olds who performed relatively poorly and the 14-year-olds who performed at adult level, leaves uncertainty about the developmental trajectory of improvements in image generation and maintenance.

In sum, it is unclear how well children and adults perform on a task requiring retrieving and maintaining known visuo-spatial images. In the current research we examined this further. The key questions were: (i) How precise are generated and maintained mental images from preschool age to adulthood (Experiment 1)? (ii). Drawing on previous work with adults focussing on the similarities between mental imagery and visual perception [1], how accurately can children coordinate visuo-spatial generated and maintained mental images with actually perceived objects and at what age do these processes become adult-like (Experiment 2)?

The challenge is to create tasks which are both suitable for young children and demand imagery in addition to visuo-spatial memory. To allow us to check that our image generation and maintenance tasks made these additional demands, visuo-spatial memory control tasks were included.

To examine developments in image generation and maintenance precision the current research used a wide age range with small intervals: Children aged 4, 6, 8, 10, and adults. Interference of task demands with imagery processes was minimised by using concrete stimuli, which have been previously shown in mental rotation tasks to be easier to visualise than abstract stimuli [26]. Moreover, drawing and image transformation tasks have shown that object familiarity and nameability affect visual remembering [21, 27]. Current tasks required, generating or maintaining an image (e.g.) of a vase rather than (e.g.) a grid pattern [23]. If young children are capable of basic image generation and maintenance then using concrete stimuli that facilitate the visualisation process may reveal early imagery competence. A further difference between our procedure and that of Kosslyn et al. [23] was that encoding time was held constant for all participants. A final modification was the type of prompt implemented before the imagery process took place. For example, for image generation participants were asked whether (e.g.) a flower was in the (imagined) vase. Importantly, this verbal prompting referred to the stimulus and did not prompt children to use mental imagery directly, thus, allowing examination of the spontaneous use of imagery. This differs from previous research that has shown that prompting the use of imagery (e.g., “make a picture in your head”) enhances performance in reasoning and problem-solving contexts in children aged 2–4 years [16–18].

In sum, the current research should provide us with insight into how spontaneous imagery develops by using concrete images, avoiding metamemorial judgements, and eliminating additional task demands such as understanding the cue or memorizing which answer-key to press.

## Experiment 1

The aim of Experiment 1 was to examine developments in image generation and maintenance. Image generation was defined as the generation of a previously presented image from memory to create a short-term visuo-spatial percept [23]. It was measured by accuracy and the time taken to judge whether a visible object (e.g., flower) “fell” onto the imagined object (i.e. vase). Image maintenance was defined as the visuo-spatial representation of an image that has been maintained in short-term memory following a delay of 500ms or 3000ms after the presentation of the image [23]. The crucial difference between image generation and maintenance was that once the image had been memorized, in generation trials participants received a 30 seconds distractor task to eliminate any short-term memory residuals, a technique commonly used in memory research [28]. In image maintenance trials no distractor task was given and the image had to be maintained for a brief time period. To explore the relation between image generation and visuo-spatial memory for the image, after the image generation and maintenance trials had been completed participants were asked to recognise the location of each object. A comparison between imagery and visuo-spatial memory performance allowed a check that the image generation and maintenance task required mental imagery and not just visuo-spatial memory.

## Method

### Participants

A total of 82 children [20 (10 males) 4-year-olds (*M* = 4.9, *SD* = 4 months), 22 (11 males) 6-year-olds (*M* = 6.9, *SD* = 3 months), 20 (10 males) 8-year-olds (*M* = 8.6, *SD* = 4 months), 20 (10 males) 10-year-olds (*M* = 10.5, *SD* = 4 months)] and 20 (10 males) adults (*M* = 21.6, *SD* = 89 months) and 9 (4 males) control adults (*M* = 22.3, *SD* = 63 months) participated. Two additional 4-year-olds were excluded because they did not complete the tasks due to inattention. In both Experiment 1 and Experiment 2, children were predominantly from a middle class background and were recruited via local primary schools, and adults were recruited from the university’s online participation system and participated in return for financial reimbursement. All children were tested following written parental consent and their own oral assent on the day of testing. Adults were tested following written consent on the day of testing. Ethical approval for this study was obtained from the Research Ethics Committee at Warwick and Plymouth University.

### Design

All participants completed both the image generation and the image maintenance tasks alongside two other imagery tasks (scanning and rotation), reported elsewhere, either in the same session (10-year-olds and adults), across 2 sessions (6- and 8-year-olds) or across 4 sessions (4-year-olds).

### Materials and Procedure

The stimuli comprised two sets of stimulus pairs, subtending between 2°-5° (height) by 2°-8° (width) of visual angle (*image generation set*: table-cake, basket-grapes, arm-watch, bowl-banana, fence-bird, vase-flower; *image maintenance set*: mountain-flag, snowman-hat, nail-hammer, tree-cherry, leaf-snail, oven-pot, ironing board-iron, squirrel-nut, swing-girl, blouse-button, chair-cat, flower-leaf). The sets were comparable in familiarity (*M* = 3.5 versus *M* = 3.8, *t*(16) < 1) and complexity (*M* = 2.6 versus *M* = 2.8, *t*(16) < 1), respectively, taken from the picture norms developed by Snodgrass and Vanderwart [29]. Both tasks were computerized and presented on a standard 17.3 inch Dell laptop PC running with the program E-Prime (www.pstnet.com; Psychology Software) that recorded the two dependent measures: (i) accuracy and (ii) time taken to respond (reaction time from stimulus onset to experimenter’s button press, see below).

Participants were required to judge whether a stimulus (e.g., flower) “fell” on a previously presented one (i.e., vase) (imagery trials) and later judged with both stimuli visible (perception control trials). Participants’ responses were a verbal “yes” or “no.” No more than three consecutive trials required the same response avoiding perseveration. Responses and reaction times were recorded with the experimenter’s right index and middle fingers pressing the “b” (yes) or “n” (no) key, respectively, immediately after a response was given. This minimised working memory demand for the participants and avoided the known risk of interference between button presses and imagery processes [30]. To check for experimenter effects, we tested 9 control adults who completed the task by pressing the buttons themselves (i.e., pressed “b” for yes responses and “n” for no responses) for both tasks. Comparing these 9 control participants who recorded their own responses with the main experimental group, there were no differences in accuracy (image maintenance: *t*(27) = .38, *p* > .05; image generation: *t*(27) = .34, *p* > .05) nor in image maintenance response time, *t*(27) = -1.14, *p* > .05, between experimenter recorded responses compared to when control participants recorded the responses themselves in image maintenance. In image generation, self-recorded responses took *longer* than experimenter-recorded responses, *t*(27) = -2.33, *p* = .03, reflecting the greater speed of verbal response. Thus, we conclude that response times have been adequately captured via the experimenter.

**Fig 1:**
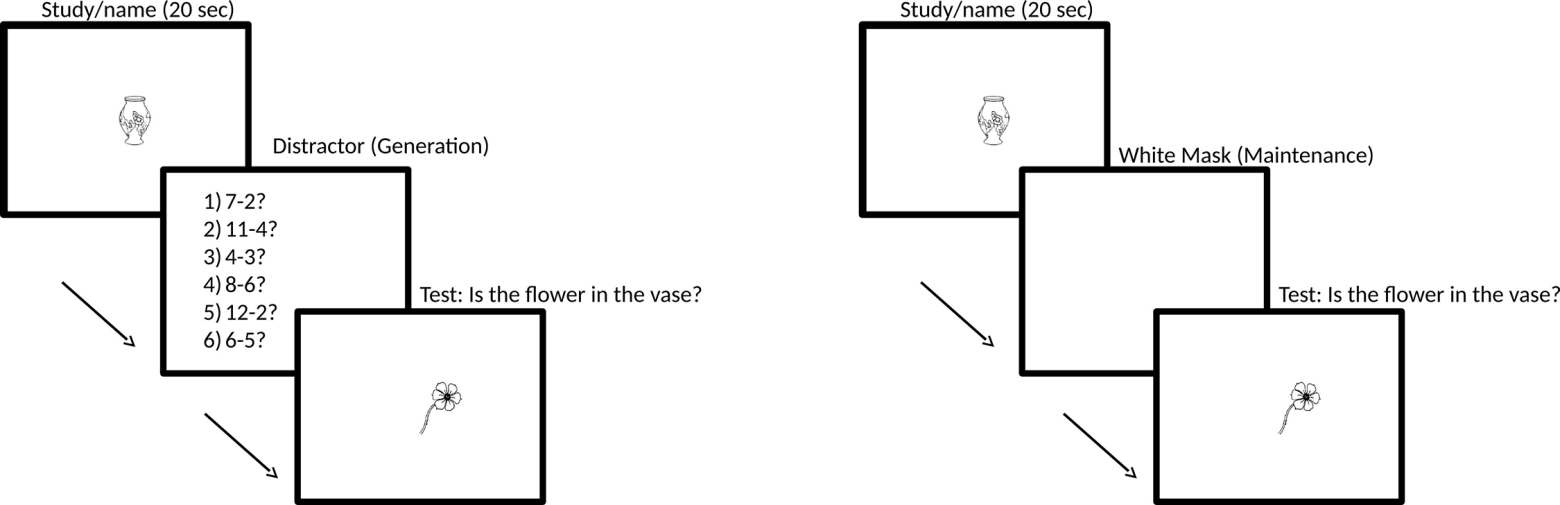
Image Generation and Maintenance task Experiment 1. Example for an image generation (30 seconds distractor) (left) and image maintenance (500ms or 3000ms white mask) (right) superimposed trial. The second image (i.e., flower) fell on the study image (i.e., vase).

Children were tested in a quiet area outside the classroom, and adults were tested in a laboratory at the university. The participants’ task was to either *generate* or *maintain* a mental image of a previously presented object accurately in its correct form, size and location on the computer screen (see Fig 1). To ensure that they understood the task, participants received two practice trials to familiarise themselves with the procedure, and younger children also received a cover story about a wizard who made pictures invisible. There were five phases for each image generation and maintenance in the following order:

1. *Study*: Participants viewed a stimulus (e.g., vase) in a specific location on the screen (x/y coordinates: 26.5mm/60mm from the closest boarders of the screen) for 20 seconds and were asked to label it. Each stimulus appeared in a different location on the screen (x/y coordinates: *Range(x)* = 3.9 to 51.5 mm/*Range(y)* = 3.9 to 102.3 mm). To enhance visuo-spatial encoding, a 3 mm wide frame appeared on the border of the screen, highlighting the border. Stimuli and frame appeared in flashing rotating rainbow colours to enhance perceptual encoding and avoid after image effects.
2. *Distractor/maintenance period*: The stimulus disappeared from the screen. In the *image generation task*, to eliminate visual short-term memory residuals and ensure that images had to be regenerated on the next phase, the participant was given a 30-second age-appropriate distractor task (counting for the 4-year-olds and mathematical calculations of increasing complexity for the older children and adults). This method is commonly applied in memory research to tap into long-term memory processes [28]. In the *image maintenance task* no distractor was given, and instead participants were simply required to retain the image for 500ms (light load) or 3000ms (heavy load) during which they were presented with a blank screen.
3. *Test (image generation or image maintenance)*: A new image (e.g., flower) appeared on the screen and participants were asked whether it fell on the original image (e.g., “Is the flower in the vase?”) that was no longer visible and thus had to be imaged.
  Phases (1)–(3) were repeated with the remaining stimuli pairs of that set, with the order of presentation randomised. Stimuli-pairs appeared in three different trial types counterbalanced between participants: i) in far trials, images appeared far from the study image (*Mdistance* = 55.3 mm), ii) in near trials images appeared close to the study image (*Mdistance* = 12.4 mm) and iii) in superimposed trials images fell on the study image. The nearer the image appeared to the study image (superimposed versus near versus far) the finer grained the generation of the shape and resolution of the image required to answer correctly. In total, there were 6 test trials for image generation (2 far, 2 near, and 2 superimposed), and 12 trials for image maintenance (6 500ms trials comprising: 2 far, 2 near, and 2 superimposed; 6 3000ms trials comprising: 2 far, 2 near, and 2 superimposed). Type of trial (near, far, superimposed) appeared in random order with each task. The participants’ answer required was “yes” or “no”. Correct answers for superimposed trials were “yes” (image generation: *N* = 2, image maintenance: *N* = 4 (2 500ms/2 3000ms)), and “no” for both near and far trials (image generation: *N* = 4, image maintenance *N* = 8 (4 500ms/4 3000ms).
4. *Memory assessment*: After all image generation or maintenance test trials had been completed participants were asked to recognize the locations of each object, assessing the memory for each original study image. Participants were presented with 4 images of the same kind (e.g., vases) next to each other on the screen (in a 2 by 2 square configuration, subtending between 3°-5° by 3°-5° of visual angle), one of which corresponded to the original studied image. The task was to select the one in the correct position. Images were presented in the same order in which they were shown earlier.
5. *Perception control*: As a final check on whether children had understood the task, perceptual control trials were administered. This phase followed exactly the same pattern as the test phase (3), except that both images were visible on the screen when the participant judged whether the second image (e.g., flower) “fell” onto the original image (i.e., vase). Results are not presented as accuracy was at ceiling for all ages.

## Results and Discussion Experiment 1

Outliers in response time, twice the mean of the age group in a particular trial (far, near, superimposed), were removed (6-year-olds: 1 data point; 10-year-olds: 3 data points). Bonferroni confidence interval adjustments and post-hoc Bonferroni analysis were used throughout.

### Preliminary analysis

The order in which image maintenance and generation tasks were completed did not affect performance (all *p*s > .05). Because there were no effects due to gender, neither within each age group nor overall (*t* < 1), gender was eliminated from subsequent analyses.

### Image generation

**Fig 2:**
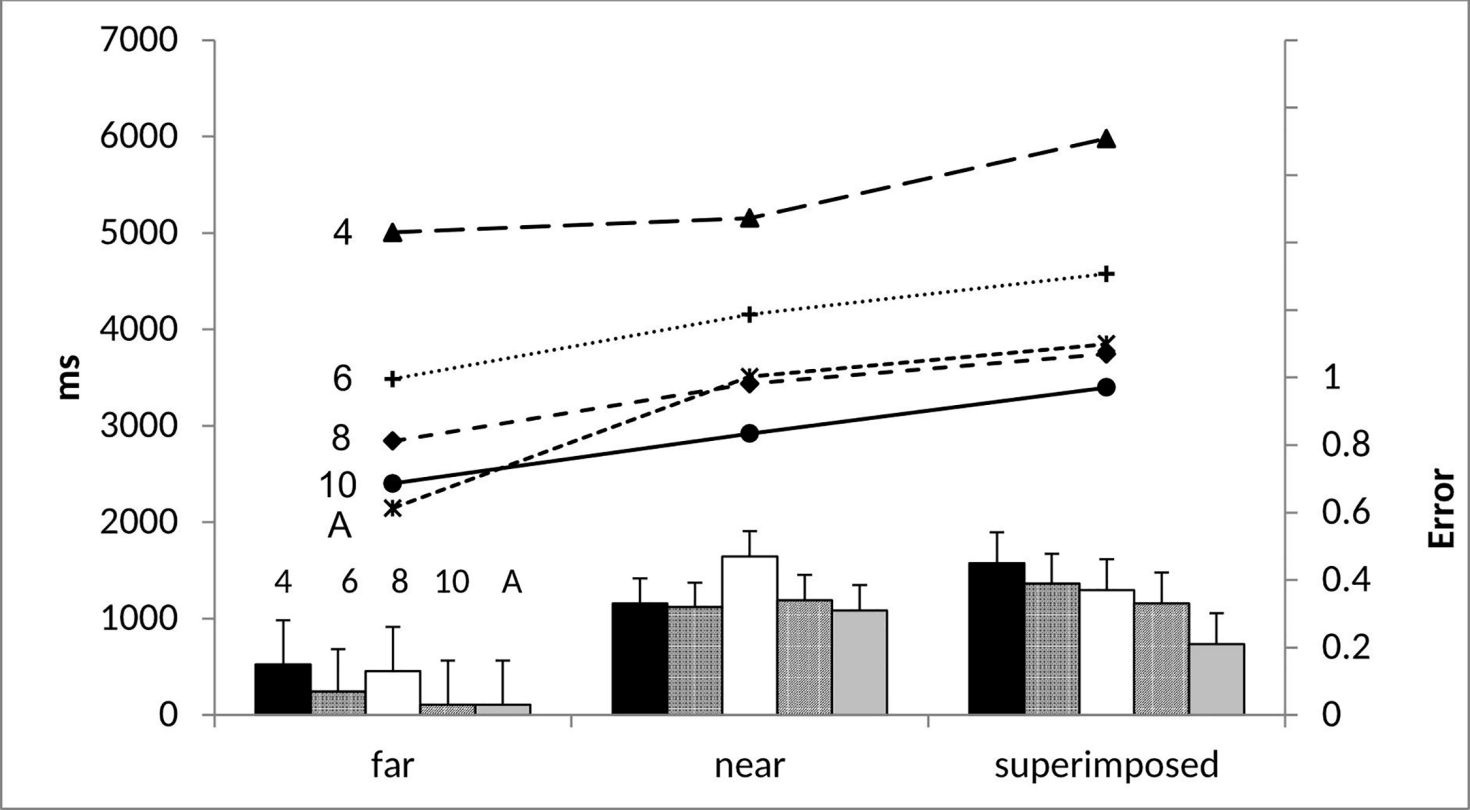
Image generation proportional error rate (bar chart) and mean response time in milliseconds (line chart) and mean standard error across all ages as a function of trial type.

Fig 2 shows the mean proportion of errors and the mean response times in the image generation trials. Image generation error rate and response time were analysed using a 5(age: 4-, vs. 6-, vs. 8-, vs. 10-year-olds, vs. adults) x 3 trial type: far vs. near vs. superimposed) analyses of variance (ANOVAs) where the first variable was between subjects and the latter within.

#### Error rate

Image generation accuracy increased with age, *F*(4, 97) = 3.35, *p* = .013, *ηp* ^*2*^ = .12, where both 4- (*M* = .69, *p* = .06 marginally significant) and 8-year-olds (*M* = .68, *p* = .02) differed from adults (*M* = .82). The remaining age groups did not differ (all *p*s > .05) (6-year-olds: *M* = .76; 10-year-olds: *M* = .77). Even 4-year-olds performed above chance (chance = .50), collapsed across superimposed, near and far trials, *t*(19) = 6.61, *p* < .001. Performance remained above chance when including only superimposed and near trials, excluding far trials, *t*(19) = 3.78, *p* = .001. This finding indicates that with minimised task demands generation abilities can be present at 4 years. The effect of trial type, *F*(2, 194) = 51.71, *p* < .001, *ηp* ^*2*^ = .35, indicated that accuracy decreased when more precision was required. Fewer errors (*p* < .001) occurred in far trials (*M* = .07) than both near (*M* = .35) and superimposed trials (*M* = .34), which did not differ. There was no trial type x age interaction, *F*(8, 194) = 1.31, *p* = .24, *ηp* ^*2*^ = .05, also indicating that even 4-year-olds understood the task instructions and their poorer performance was not due to the nature of task.

#### Response time

The time taken to generate a mental image decreased with age, *F*(4, 96) = 32.50, *p* < .001, *ηp* ^*2*^ = .58, between all adjacent age groups (all *p*s < .001) until 8 years, where the oldest three groups did not differ (*p* > .05) (4-year-olds: *M* = 5413ms; 6-year-olds: *M* = 4071ms; 8-year-olds: *M* = 3340ms; 10-year-olds: *M* = 2906ms; adults: *M* = 3167ms). Four-year-olds’ response time was about half that of 5-year-olds in previous research (approximately 10 seconds) [23]. Thus, verbally reporting the product of imagery significantly reduces response time and highlights that Kosslyn et al’s [23] response time results may not directly reflect the time it took to generate an image but additional task factors such as remembering which button to press interfering with imagery [30]. The closer the image appeared to the visible stimulus the longer it took to respond, *F*(2, 192) = 37.70, *p* < .001, *ηp* ^*2*^ = .28, and this was the case for all ages as there was no trial x age interaction, *F*(8, 192) = 1.53, *p* = .15, *ηp* ^*2*^ = .06. Post-hoc analyses show that superimposed trials took the longest in each age group, however, differences between trial types are not exactly the same across age groups. Specifically, 4-year-olds took longer to respond in superimposed than both near and far trials (*p*s < .036), 6-year-olds took longer in superimposed than far trials (*p* = .003) and near than far trials (*p* = .02), 8-year-olds took longer in superimposed than far trials (*p* = .028) and marginally longer in near than far trials (*p* = .063), 10-year-olds took longer in superimposed than far trials (*p* = .016) and adults took longer in superimposed than far trials (*p* < .001) and near than far trials (*p* < .001). There were no further differences. The comparable effects of complexity on response time and error rate rule out a speed-accuracy trade off.

#### Visuo-spatial memory versus image generation

How well was the original image remembered? All age groups performed above chance (*p* < .001) in selecting the correct study image out of 4 presented images (one-sample t-tests, chance = .25). Although performance increased with age, *F*(4, 97) = 7.41, *p* < .001, *ηp* ^*2*^ = .23, this effect was due to significantly poorer performance (all *p*s < .01) by 4-year-olds (*M* = .55) than older participants (ranging from *M* = .77 at 6 years to *M* = .87 for adults) who did not differ (all *p*s > .05). This contrasts with image generation performance, which continued to increase with age, suggesting that processes beyond visuo-spatial memory are involved in the generation of a mental image.

To directly examine the link between memory and image generation, correlations were performed for the youngest 3 age groups only, a10-year-olds and adults performed at ceiling. No association emerged between image generation accuracy and corresponding memory accuracy overall for the youngest 3 age groups, *r* = .18, *p* = .17 and when examining associations for each age group individually: 4-year-olds: *r* = .04; 6-year-olds: *r* = .35, 8-year-olds: *r* = -.24, all *p*s > .05. Again, this suggests that poor image generation is not a result of poor visual memory.

### Image Maintenance

**Fig 3:**
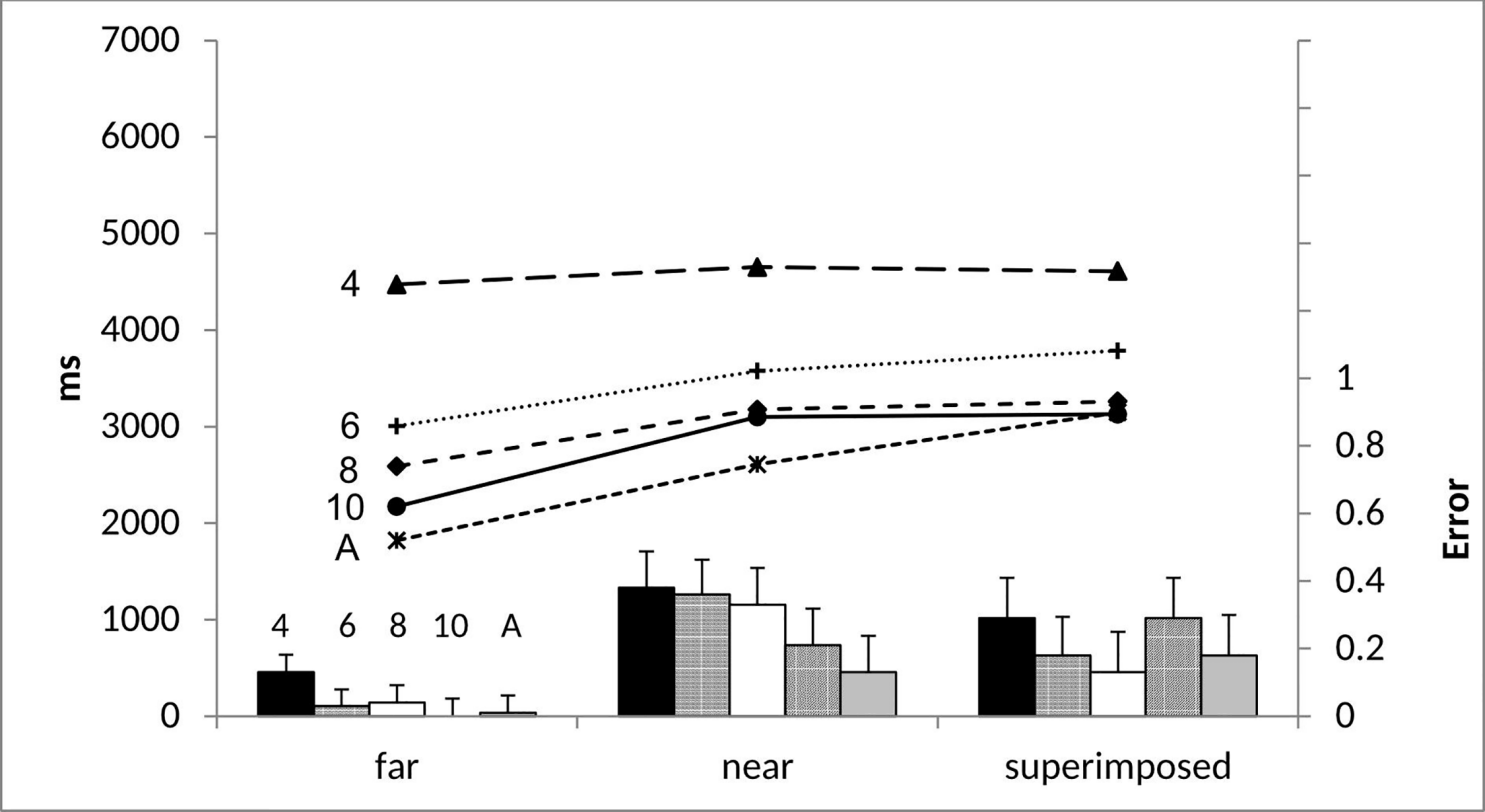
Image maintenance proportional error rate (bar chart) and mean response time in milliseconds (line chart) and mean standard error across all ages as a function of trial type.

Fig 3 shows the mean proportion of errors and mean response times in the image maintenance trials. Image maintenance proportional error rate and response time were analysed in two 5(age: 4-, vs. 6-, vs. 8-, vs. 10-year-olds, vs. adults) x 3(trial type: far vs. near vs. superimposed) x 2(load: 500ms vs. 3000ms) ANOVAs. As there were no effects of maintenance load (500ms vs. 3000ms) for accuracy, *F*(1, 194) = .01 *p* = .98, *ηp* ^*2*^ = .00, and for response time, *F*(1, 194) = 2.11, *p* = .15, *ηp* ^*2*^ = .02, this variable was eliminated from subsequent analyses. The means of both imagery loads’ error rates and response times were used for further analyses.

#### Error rate

Image maintenance accuracy increased with age *F*(4, 97) = 5.63, *p* < .001, *ηp* ^*2*^ = .19, particularly between 4-year-olds (*M* = .74) and all older age groups (8-year-olds: *M* = .84, *p* = .04; 10-year-olds: *M* = .83, *p* = .06 marginally significant; adults: *M* = .90, *p* < .001) except 6-year-olds (*M* = .81, *p* > .05). There were no other age group differences (*p*s > .05). However, even 4-year-olds performed significantly above chance, *t*(19) = 7.75, *p* < .001. Performance remained above chance when including only superimposed and near trials, excluding far trials, *t*(19) = 4.48, *p* < .001. Further, accuracy was better for far versus near or superimposed trials, *F*(2, 194) = 27.79, *p* < .001, *ηp* ^*2*^ = .22. There was no interaction between trial type and age (*p* > .05).

#### Response time

Response time decreased with age, *F*(4, 97) = 27.81, *p* < .001, *ηp* ^*2*^ = .53, where 4-year-olds (*M* = 4578ms) differed (all *p*s < .001) from all older age groups (6-year-olds: *M* = 3455ms; 8-year-olds: *M* = 3009ms; 10-year-olds: *M* = 2799ms; adults: *M* = 2525ms) and 6-year-olds took longer than both 10-year-olds (*p* = .03) and adults (*p* < .001). There were no further differences. Importantly, response times were affected by trial type; *F*(2, 194) = 17.57, p = .001, ηp^*2*^ = .15, in that participants responded faster in far trials compared to near and superimposed trials which did not differ (*M* = 2848ms versus *M* = 3386ms *M* = 3586ms, *p* < .001). Again, the superior performance on far trials suggests that mental imagery processes go beyond the time it takes to visually inspect a stimulus. The comparable effects of trial complexity on response time and error rate also rule out speed-accuracy trade off.

#### Visuo-spatial memory versus image maintenance

One-sample t-tests indicated that each age group performed above chance (*p*s < .001) in selecting each study image out of 4 presented images (chance = .25). Four-year-olds (*M* = .62) memorized fewer study images (ranging from *M* = .77 for 6-year-olds to *M* = .81 for adults, all *p*s < .05), *F*(4, 96) = 6.08, *p* < .001, *ηp* ^*2*^ = .20, and there were no further differences between age groups (*p*s > .05). As above, there were no correlations between memory accuracy and the corresponding maintenance accuracy overall for the youngest 3 age groups, *r* = .23, *p* = .07 (after partialling out age, *r*_*partial*_ = .10, *p* = .45) and when examining associations for each age group individually (4-year-olds: *r* = .01, 6-year-olds: *r* = .03, and 8-year-olds: *r* = .31, all *p*s > .05).

### Image generation versus image maintenance performance

To directly compare error rates and response times in image generation and maintenance two 5(age: 4-, vs. 6-, vs. 8-, vs. 10-year-olds, vs. adults) x 2(task: image generation vs. image maintenance) repeated measures ANOVAs were conducted. For error rates, there was a main effect of age, *F*(4, 97) = 6.21, *p* < .001, *ηp* ^*2*^ = .20, where both 4-year-olds (*M* = .72, p < .001) and 8-year-olds (*M* = .76, p = .01) differed from adults (*M* = .86) but not from 6-year-olds (*M* = .78) and 10-year-olds (*M* = .79). There were no further age group differences (*p*s > .05). There was also a main effect of task, *F*(1, 97) = 21.81, p < .001, *ηp* ^*2*^ = .18, indicating that image generation (*M* = .74) was harder than image maintenance (*M* = .82). There was no age x task interaction, *F*(4, 97) = 1.49, *p* = .21, *ηp* ^*2*^ = .06, but when examining differences for each age group separately, then only the oldest three age groups performed significantly worse in image generation than maintenance (*p*s < .05).

For response times there was also a main effect of age, *F*(4, 96) = 42.63, *p* < .001, *ηp* ^*2*^ = .64, where 4-year-olds (*M* = 4995ms) took longer to respond than all older age groups (all *p*s < .001). Six-year-olds (*M* = 3763ms) took also longer than all older age groups (all *p*s < .025). The oldest 3 age groups did not differ (all *p*s > .05) (8-year-olds: *M* = 3175ms; 10-year-olds: *M* = 2864ms; adults: *M* = 2846ms). Additionally, image generation took longer (*M* = 3779ms) than image maintenance (*M* = 3278ms), *F*(1, 96) = 37.64, *p* < .001, *ηp* ^*2*^ = .28. However, these effects were qualified by an age x task interaction, *F*(4, 96) = 2.52, *p* = .046, *ηp* ^*2*^ = .09. All age groups (all *p*s < .001; except 8-year-olds: *p* = .07, marginally significant), except 10-year-olds (*p* > .05) took longer in image generation than image maintenance trials; the lack of difference for 10-year-olds alone may not be a reliable phenomenon.

The overall task performance differences suggest that additional processes are required for generation compared to maintenance. Both processes require activating stored visual memories, but image generation may additionally require accessing stored visual memories for information about what to image [23]. Our findings of higher error rate in the three oldest age groups and longer response times in image generation support this claim.

In sum, the results of Experiment 1 suggest that basic image generation and maintenance abilities can be present at 4 years of age. That is, in addition to research finding that 2- to 4-year-old children use imagery when prompted to do so (e.g., “make a picture in your head”) [16–18] the current research reveals early spontaneous imagery abilities. Additionally, the precision with which images are generated and maintained improves particularly between 4 and 8 years of age. Moreover, mental imagery processes go beyond the time it takes to visually inspect a stimulus for all ages, shown by the finding that response time was shorter in far trials compared to both near and superimposed ones. Finally, the lack of a correlation between imagery and according visuo-spatial memory may suggest that visuo-spatial memory is not required for both image generation and maintenance tasks. Alternatively, it may indicate that visuo-spatial recognition may lack sensitivity and not be the most suitable comparator to image generation and maintenance, see [31, 32] for associating recall with recognition. Therefore, in Experiment 2 a measure of visuo-spatial recall was introduced.

## Experiment 2

Experiment 1 examined the precision with which images are generated and maintained. The aim of Experiment 2 was to compare the ability to coordinate mental and real images. Comparing coordinating a mental image and a visual image with children’s ability to coordinate two visual images should provide a very accurate assessment of their mental imagery abilities, holding other task factors constant. Image generation and maintenance were measured by the precision with which a second visible object was manipulated within the context of the first imagined object (imagery trials), compared to how the second object was manipulated when the first image was also visible (perception control trials). As in Experiment 1, the crucial difference between image generation and maintenance was that once the image had been memorized, in generation trials participants received a 30 seconds distractor task in order to eliminate any short-term memory residuals [28]. In image maintenance trials there was no distractor task and the image had to be maintained for 3000ms.

Additionally, as before visuo-spatial memory was assessed. Instead of recognising the location of each object (Experiment 1), in the current experiment participants recalled the location of each object by dragging and dropping the stimulus onto its original place. Examination of an objects’ dropped location compared to its original actual location provided precise measures of visuo-spatial location memory. Further, comparison between imagery and visuo-spatial memory performance allowed a check that the image generation and maintenance tasks required mental imagery and not just visuo-spatial memory.

## Method

### Participants

A total of 88 children [23 (12 males) 4-year-olds (*M* = 4.8, SD = 4 months), 21 (10 males) 6-year-olds (*M* = 6.7, SD = 3 months), 22 (11 males) 8-year-olds (*M* = 8.8, SD = 3 months), 22 (11 males) 10-year-olds (*M* = 10.9, SD = 3 months) and 21 (10 males) adults (*M* = 23.6, SD = 84 months)] were recruited. All children were tested following written parental consent and their own oral assent on the day of testing. Adults were tested following written consent on the day of testing. Ethical approval for this study was obtained from the Research Ethics Committee at Warwick and Plymouth University.

### Design

All participants completed both the image generation and the image maintenance tasks in the same session, with task order counterbalanced within each age group.

### Materials and Procedure

The stimuli comprised two sets of 6 pairs of stimuli taken from those used in Experiment 1 and each participant completed 12 trials. As in Experiment 1 the program recorded response times (reaction time from stimulus onset to pressing the return key) and imagery-perception overlap (as described below) and memory scores.

**Fig 4:**
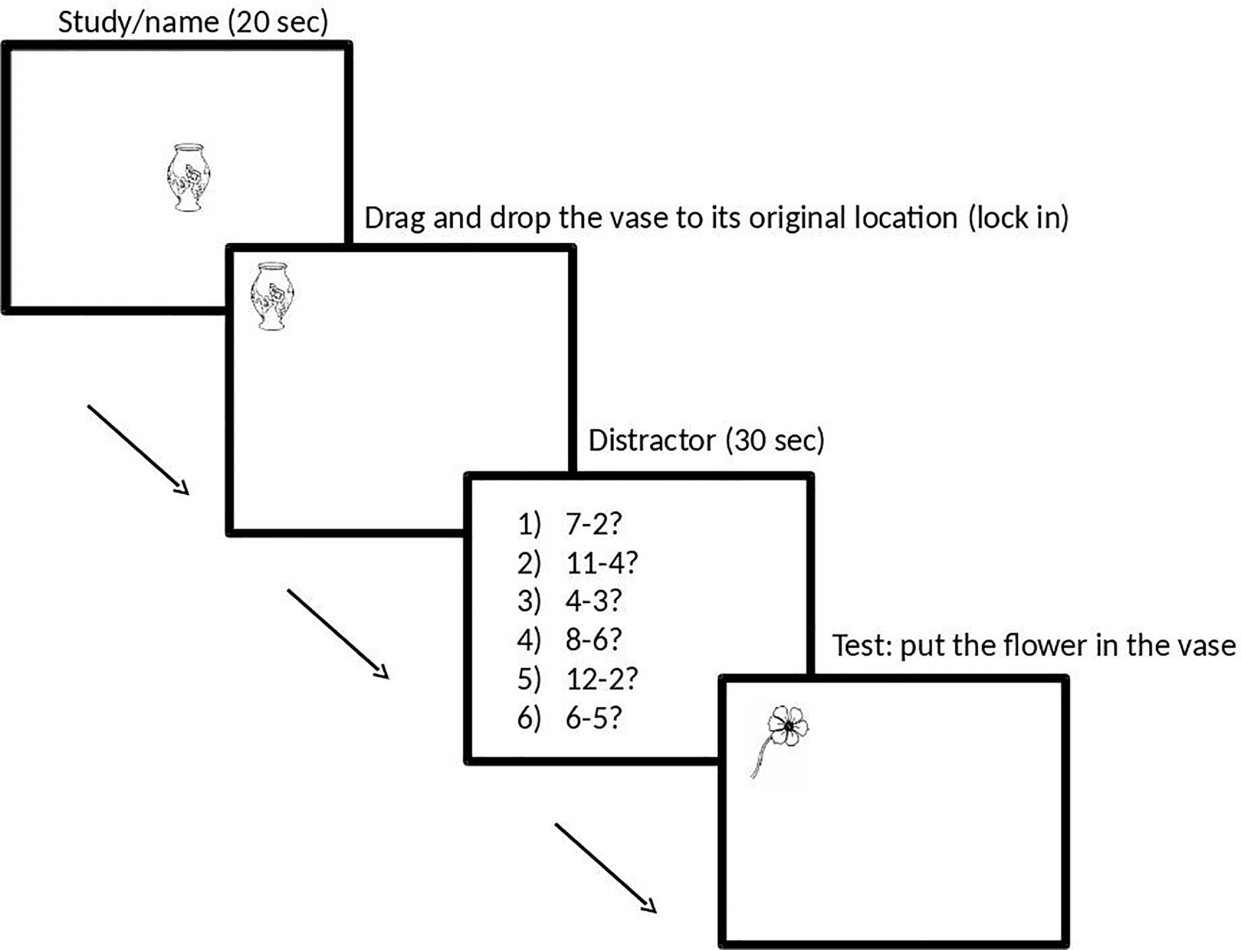
Image Generation and Maintenance task Experiment 2. Example of an image generation trial. Image maintenance followed the same pattern except that a blank screen was presented for 3000ms instead of the distractor task.

The procedure followed the same pattern as Experiment 1 with two modifications (Fig 4). First, instead of judging whether a presented stimulus fell on an imaged one, participants were required to drag and drop a visible stimulus onto an imaged one (imagery trials), before later dragging and dropping the same stimulus, but this time with both stimuli being visible (perception control trials). Because some of the ‘correct’ positions for the stimuli were subjective (e.g., the flower could be placed on top of the vase, or it could be placed further inside the vase; both positions would be correct), overlap scores were calculated, using Pythagoras’ theorem (h^2^ = a^2^ + b^2^), as the difference (in pixels) between the centre of the object (defined as the centre of a non-visible box drawn around it) being moved on the imagery trials and its centre point on the perception control trials. Hence, participants determined their own individual baseline positions for the stimuli in the perception control trials. Second, in the memory trials, rather than recognizing the imaged objects’ correct location, participants recalled spatial location by dragging and dropping the object back in its original place. Memory accuracy for each image was calculated as the distance (in pixels) from the centre point of the original image location to the centre point of the dropped image location (again using Pythagoras theorem).

## Results and Discussion Experiment 2

Outliers in response time and overlap (i.e. two standard deviations above the mean) of the according age group were removed (only one 4-year-old was removed as a whole participant as more than half of his data points were outliers and there were no further outlier data points). Bonferroni confidence interval adjustments and post-hoc Bonferroni analysis were used throughout. ‘Overlap’ refers to the difference in pixels between the location a picture was placed on a real image (in the perception control trials) and on an imagined image (in the image generation/maintenance trials). Lower scores indicate greater similarity between accuracy using mental images and real pictures.

### Preliminary analyses

There was a main effect of gender for image maintenance overlap, *F*(1, 99) = 4.53, *p* < .05, *ηp* ^*2*^ = .04. For females, accurate placement of a picture on a real image and on a mental image was more similar than for males *(M* = 133.13 versus *M* = 143.45). However, there was a marginally significant age x gender interaction for image maintenance overlap, *F*(4, 99) = 2.15, *p* = .08, *ηp* ^*2*^ = .08, due to the fact this gender difference was significant only for 4-year-olds (*p* < .05), and not for any of the older age groups (all *p*s > .12).

### Image generation

**Fig 5:**
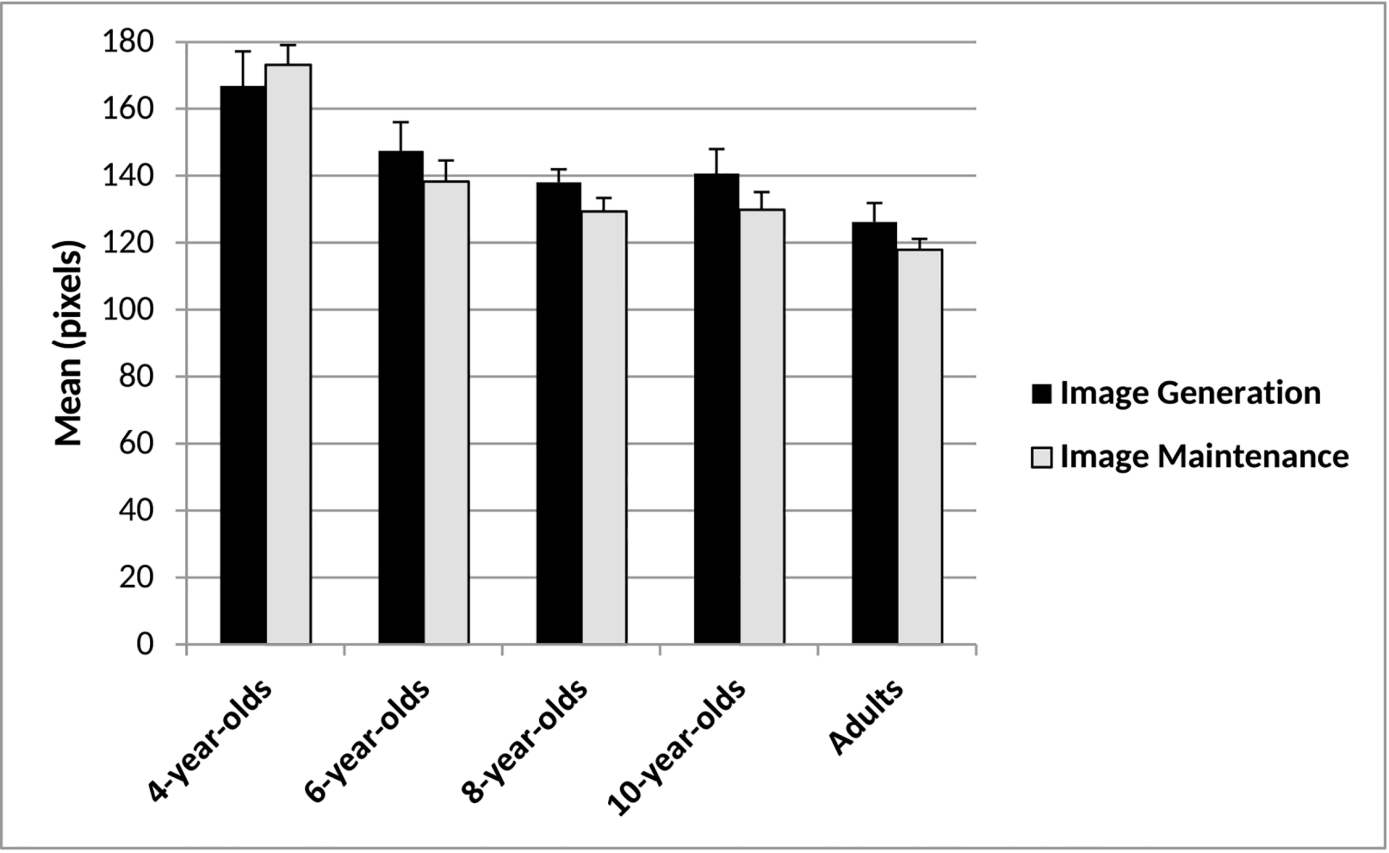
Image generation and image maintenance overlap scores and mean standard error for each age group.

**Fig 6:**
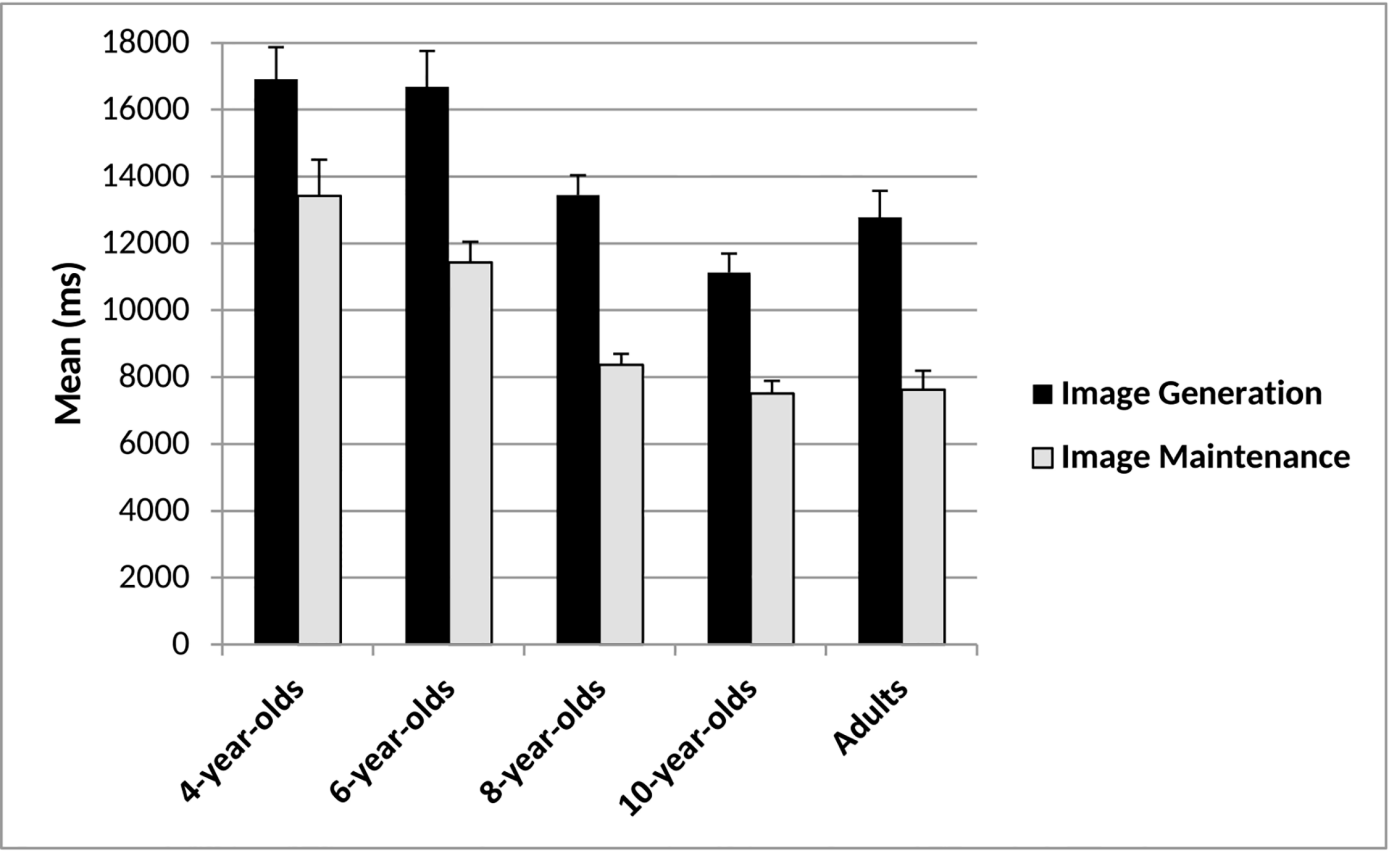
Mean response times for image generation and image maintenance tasks and mean standard error for each age group.

Figs 5 and 6 display age-related differences in image generation abilities.

### Overlap generation-perception

There was a significant effect of age for image generation overlap, *F*(4, 103) = 3.97, *p* < .005, *ηp* ^*2*^ = .13. Adults’ placement of a picture on a real image and on a mental image overlapped more (*M* = 126.25) than 4-year-olds (*M* = 166.81, *p* < .005). However there were no significant differences between adults and children older than 4 years (all *p*s > .52), indicating that children could generate adult-like mental images in this task by age 6.

### Response time generation-perception

To examine age-related differences in processes underlying image generation compared to those involved in visual inspection and motor coordination (dragging and dropping of a stimulus using the computer mouse), image generation and perception control response times were compared. A 2(trial: generation vs. perception) x 5(age: 4- vs. 6- vs. 8- vs. 10-year-olds vs. adults) ANOVA showed main effects of age, *F*(4, 103) = 16.09, *p* < .001, *ηp* ^*2*^ = .39, where 4-year-olds (*M* = 14428m) took equally long as 6-year-olds (*M* = 13394ms, *p* > .05) and were slower than all older age groups (all *p*s < .001). Additionally, 6-year-olds took longer than 8-year-olds (*M* = 10063ms, *p* = .007), 10-year-olds (*M* = 8249ms, *p* < .001) and adults (*M* = 9204, *p* < .001). Children from age 8 onwards did not differ (p > .05). Further, image generation trials took markedly longer (*M* = 14187ms) than perception control trials (*M* = 7945ms), *F*(1, 103) = 362.83, *p* < .001, *ηp* ^*2*^ = .78, suggesting image generation processes go beyond visual inspection of a stimulus and the motoric components for all age groups. There was no age x trial interaction, *F*(4, 103) = 1.44, *p* = .23, *ηp* ^*2*^ = .05.

### Visuo-spatial memory versus generation overlap

Memory scores were calculated in terms of overlap as before. Lower scores reflect more accurate memory. A one-way ANOVA revealed a significant main effect of age, *F*(4, 103) = 11.60, *p* < .001, *ηp* ^*2*^ = .31. Four-year-olds remembered the image locations significantly (all *p*s < .001) less accurately (*M* = 157.14) than each of the other age groups (overlap scores ranged from *M* = 100.00 for 6-year-olds to *M* = 78.19 for adults). There were no further significant differences (all *p*s > .05).

To explore whether image generation overlap depends on how well the visuo-spatial locations of the images are remembered, we conducted a correlational analysis between memory accuracy and image generation overlap. There was a strong relation between memory accuracy and generation overlap, *r* = .38, *p* < .001, that held when controlling for age, *r* = .32, *p* = .001, suggesting that spatial memory is required for this visuo-spatial image generation task and *vice versa*.

### Image maintenance

Figs 5 and 6 display developmental differences in image maintenance abilities.

### Overlap maintenance-perception

There was a main effect of age for image maintenance overlap, *F*(4, 104) = 17.90, *p* < .001, *ηp* ^*2*^ = .41. Four-year-olds (*M* = 173.19) maintained images had significantly less overlap than those of older children (overlaps ranged from *M* = 138.26 for six-year-olds to *M* = 117.96 for adults; the difference between 6-year-olds and adults was marginally significant, *p* = .06). Thus, at least by 8-years-of-age children maintained images as precisely as adults in this task (Fig 5).

### Response time maintenance-perception

To examine developmental processes underlying image maintenance beyond processes underlying visual inspection and motor coordination, image maintenance and perception control response times were compared. A 2 (trial: maintenance vs. perception) x 5(age: 4- vs. 6- vs. 8- vs. 10-year-olds vs. adults) ANOVA showed decreasing response time with increasing age, *F*(4, 103) = 23.29, *p* < .001, *ηp* ^*2*^ = .47, where 4-year-ods (*M* = 12446ms) took equally long as 6-year-olds (*M* = 10435ms, *p* > .05) but longer than all older age groups (all *p*s < .001). Six-year-olds took also longer than 8- (*M* = 7511ms, *p* = .004), 10-year-olds (*M* = 6158ms, *p* < .001) and adults (*M* = 6721ms, *p* < .001), who did not differ from each other (*p* > .05). Further, image maintenance trials (*M* = 9668ms) took longer than perception trials (*M* = 7640ms), *F*(1, 104) = 65.88, *p* < .001, *ηp* ^*2*^ = .39. There was no interaction, *F*(4, 104) = .51, *p* = .73, *ηp* ^*2*^ = .02. Findings suggest that image maintenance processes go beyond visual inspection of a stimulus and the motoric components of the task for all ages.

### Visuo-spatial memory versus maintenance overlap

There was a main effect of age group for memory accuracy, *F* (4, 104) = 12.09, *ηp* ^*2*^ = .32. Four-year-olds had significantly less accurate memory for the images (*M* = 156.69) than did older participants (ranging from *M* = 93.20 for 6-year-olds to *M* = 74.60 for adults, all *p*s < .001). There were no differences in memory accuracy between any of the older age groups within whom there were no significant differences (*p* > .05). Moreover, there was a significant relation between image maintenance performance and memory accuracy, *r* = .55, *p* < .001, that held when controlling for age, *r*_*partial*_ = .49, *p* < .001, suggesting that spatial memory is associated with image maintenance processes and *vice versa*.

### Image generation versus image maintenance performance

We compared precision on the image generation and image maintenance tasks with a 5(age group: 4-, vs. 6-, vs. 8-, vs. 10-year-olds, vs. adults) x 2(task: image generation vs. image maintenance) repeated measures ANOVA. There was a main effect of age for overlap, *F*(4, 103) = 16.51, *p* < .001, *ηp* ^*2*^ = .39, where 4-year-olds (*M* = 169.78) performed less accurately (all *p*s < .001) than all older age groups (6-year-olds: *M* = 142.84; 8-year-olds: *M* = 133.74; 10-year-olds: *M* = 135.29; adults: *M* = 122.11) and 6-year-olds performed less accurately (*p* = .01) than adults. There were no further age group performance differences (*p*s > .05). Additionally, overlap did not differ between image generation (*M* = 143.98) and image maintenance (*M* = 137.83), *F*(1, 103) = 2.20, *ηp* ^*2*^ = .02 and there was no significant age x task interaction, *F*(4, 103) = .55, *p* = .70, *ηp* ^*2*^ = .02 (Fig 5).

For response times a 5(age group) x 2(task) ANOVA yielded significant main effects of age, *F* (4, 103) = 14.16, *ηp* ^*2*^ = .36, where 4-year-olds (*M* = 15000ms) performed as fast as 6-year-olds (*M* = 14053ms, *p* > .05) but slower than all older age groups (all *p*s < .001). Six-year-olds took also longer than all older age groups (8-year-olds: *M* = 10906ms, *p* = 0.11; 10-year-olds: *M* = 9322ms, *p* < .001; and adults: *M* = 10196ms, *p* = .001). The oldest three age groups did not differ (all *p*s > .05). Moreover, participants were faster to respond on the image maintenance task (*M* = 9605ms) than they were on the image generation task (*M* = 14177ms), *F*(1, 103) = 236.37, *p* < .001, *ηp* ^*2*^ = .70. There was no interaction, *F*(4, 103) = 1.44, *p =* .23, *ηp* ^*2*^ = .05, indicating that all age groups were slower to respond on the image generation task compared to the maintenance task (Fig 6). Thus, as in Experiment 1, response time differences may reflect the time taken accessing long-term memory for information about what to image (image generation) in addition to holding an image online (image maintenance).

## General Discussion

This research aimed to examine the precision with which children from 4 years up to adulthood generate and maintain mental images (Experiment 1) and their accuracy coordinating mental images compared to visually perceived objects (Experiment 2). Findings from Experiment 1 suggest that the precision with which images are generated and maintained undergoes significant development over the preschool period. Moreover, although 4-year-olds’ accuracy was above chance in both image generation and maintenance in Experiment 1, from 4- to 8-years-of-age, children’s ability to coordinate mental and real images becomes increasingly similar to adults’ (Experiment 2).

By modifying methods from Kosslyn et al. [23], who reported that 5- and 8-year-old children were significantly worse at generating and maintaining mental images than 14-year-old children, we have shown that, children as young as 4 can generate and maintain mental images. Hence our findings are more in-line with research that reported even 3-year-olds are capable of using mental imagery for reasoning and problem solving, although they tend not to do so spontaneously and require instructions and support in order to use their imagination [17, 18, 33]. Importantly, what the current findings add is that not only can young children use mental imagery when instructed to do so but both their accuracy in a task requiring image generation and maintenance and the ability to coordinate real with imaged images increases with age, with 4-year-olds showing basic imagery abilities.

Age-related advances in image maintenance abilities that we observed occur at an earlier age than reported improvements in visuo-spatial working memory span. Image maintenance may be regarded as a critical function of visuo-spatial working memory [34]. Thus, improvements on image maintenance may precede processes required for visuo-spatial working memory span tasks. Studies that ask children to remember the locations of filled in squares or dots within an image or a square, for example, report that 5-year-olds perform at approximately 25% of adult-level [35], and that visual and spatial working memory span continue to improve from age 5 through to 11 years [36, 37, 38]. Kail [8] argues that mental imagery actually predicts spatial memory span, and thus improvements in mental imagery may facilitate improvements in spatial memory abilities. The current finding of developments in image maintenance over preschool prior to reported later developments in visuo-spatial working memory span [38] and the relation between imagery performance and memory performance (Experiment 2) may support this claim.

In contrast to image maintenance which relies on short-term memory, image generation involves generating a previously seen image from long-term memory. Kosslyn et al.’s [23] measure of image generation was the time taken to retrieve an image from long-term memory. Their task required children to generate an upper-case version when cued with a lower-case letter, while in our study participants were cued verbally to imagine a previously presented image. Both of these measures of image generation require that participants generate an image from memory. Both also require generating reproductive images, a term first used by [20], that is, evoking images for objects or events that are already known, which is possible at a younger age than transformed images. Future work should extend the present methodologies to examine the developmental trajectory of transformed image abilities, and at which age children can produce these types of images with adult-like precision.

Can we rule out the possibility that the present tasks are measures of visuo-spatial memory rather than visuo-spatial mental imagery? To explore the relation between imagery and spatial memory for the image, after the imagery trials had been completed we asked participants to recognize and recall the spatial locations of each of the images. Memory trials were examined after all imagery trials, yet even after this relatively long period, performance on the visuo-spatial recognition and recall tasks remained high. In Experiment 1 there was no relation between imagery accuracy and visuo-spatial recognition. However, the comparison of recognition (visuo-spatial memory) and recall (image generation and maintenance) may not have been appropriate [31]. Our finding that visuo-spatial recall (Experiment 2) correlated with the ability to coordinate a visible image with an imaged one supports this and suggests that the difficulty of the image generation and maintenance tasks lies in visuo-spatial memory. Weakly stored visuo-spatial locations may have contributed to poorer imagery performance. That is, for our visuo-spatial imagery tasks a lack of spatial memory may make it difficult to impose an image onto the imagined object, even if the shape and resolution of the imagined object were generated precisely. However, the findings of different developmental trajectories for each imagery task and visuo-spatial memory task suggest that the imagery tasks demand more than visuo-spatial memory. Specifically, in both visuo-spatial recognition and recall tasks only 4-year-olds differed from each of the age groups whereas image generation and maintenance performance continued to improve with age, particularly between 4- and 8 years. Thus, memory of the spatial location is necessary but not sufficient to generate and maintain the correct size and resolution of an image.

The current research also adds to our understanding of development of spatial cognition. Research on visuo-spatial cognition indicates that basic spatial coding abilities are present in infancy [39]. However, location memory undergoes significant changes from toddlerhood into adulthood. Geometric biases in how children categorise space occur when younger children form large geometric categories and are biased towards the centre of the space (e.g., a midline) while older children subdivide space into smaller regions, showing biases away from a category boundary at midline [40, 41]. For example, 7-year-old children replace objects into their original locations with significantly less accuracy than older children and adults, suggesting increasing precision in metric location estimation [42]. The current findings suggest that additionally, on a local level, the precision with which the shape and resolution of remembered visual stimuli are generated and maintained in their visuo-spatial location undergoes significant changes over the primary school period. Moreover, the extent of the accuracy of coordinating mental images compared to visual images increases particularly over the preschool period.

Research on encoding spatial object relations, suggests that even infants are able to encode basic spatial relations between objects [43] and by 4 years children encode extent in absence of frame of reference [44, 45]. The current findings add that encoding spatial relations is also possible between mental and visual images.

In sum, this is the first investigation to demonstrate basic image generation and maintenance abilities in 4-year-olds. Importantly, the precision with which they generate and maintain visuo-spatial mental images undergoes significant developments over the early primary school period. Moreover, children’s mental images can be accurately coordinated with visually perceived objects. Thus, in addition to previous research that has shown that mental imagery aids cognitive functioning, the current research suggests that children can accurately generate and maintain their remembered visuo-spatial mental images and coordinate mental and visually perceived images in preschool age.
